# Species turnover within cystic fibrosis lung microbiota is indicative of acute pulmonary exacerbation onset

**DOI:** 10.1186/s40168-025-02143-5

**Published:** 2025-06-07

**Authors:** Leah Cuthbertson, Lauren Hatfield, Helen Gavillet, Michelle Hardman, Ryan Marsh, Damian W. Rivett, Christopher van der Gast

**Affiliations:** 1 Department of Respiratory Science, University of Leicester, Leicester, UK; 2 Department of Life Sciences, Manchester Metropolitan University, Manchester, UK; 3 Department of Applied Sciences, Northumbria University, Newcastle, UK; 4 Swansea University Medical School, Swansea University, Swansea, UK; 5 Department of Natural Sciences, Manchester Metropolitan University, Manchester, UK; 6 Department of Respiratory Medicine, Northern Care Alliance NHS Foundation Trust, Salford, UK

**Keywords:** Cystic fibrosis, Lung microbiome, Temporal dynamics, Island biogeography, Species-time relationships, Microbiome ecology, Species turnover, Lung function, Pulmonary exacerbation

## Abstract

**Background:**

Acute pulmonary exacerbations (PEx) are associated with increased morbidity and earlier mortality for people living with cystic fibrosis (pwCF). The most common causes of PEx in CF are by bacterial infection and concomitant inflammation leading to progressive airway damage. To draw attention to the seriousness of PEx they have been labelled as ‘lung attacks’, much like a ‘heart attack’ for acute myocardial infarction. Treatment typically starts when a pwCF presents with worsening respiratory symptoms. Hence, there is a pressing need to identify indicative biomarkers of PEx onset to allow more timely intervention. Set within an ecological framework, we investigated temporal microbiota dynamics to connect changes in the lung microbiota of pwCF to changes in disease states across a PEx event.

**Results:**

Species-time relationships (STR) describe how the richness of a community changes with time, here STRs were used to assess temporal turnover (*w*) within the lung microbiota of each pwCF (*n* = 12, mean sample duration 315.9 ± 42.7 days). STRs were characterised by high interpatient variability, indicating that turnover and hence temporal organization are a personalized feature of the CF lung microbiota. Greater turnover was found to be significantly associated with greater change in lung function with time. When microbiota turnover was examined at a finer scale across each pwCF time series, *w*-values could clearly be observed to increase in the exacerbation period, then peaking within the treatment period, demonstrating that increases in turnover were not solely a result of perturbations caused by PEx antibiotic interventions. STR *w*-values have been found to have a remarkable degree of similarity for different organisms, in a variety of habitats and ecosystems, and time lengths (typically not exceeding *w* = 0.5). Here, we found *w*-values soon increased beyond that. It was therefore possible to use the departure from that expected norm up to start of treatment to approximate onset of PEx in days (21.2 ± 8.9 days across the study participants).

**Conclusions:**

Here, we illustrate that changes in turnover of the lung microbiota of pwCF can be indicative of PEx onset in considerable advance of when treatment would normally be initiated. This offers translational potential to enable early detection of PEx and consequent timely intervention.

Video Abstract

**Supplementary Information:**

The online version contains supplementary material available at 10.1186/s40168-025-02143-5.

## Background

Cystic fibrosis (CF) is characterised by a vicious cycle of recurring respiratory infection and consequent inflammation that gradually damages lung tissue and degrades lung function throughout the life of a person with CF (pwCF) [1, 2]. Additionally, that cycle is further intensified by the frequency and cumulation of acute pulmonary exacerbations (PEx) experienced through the life of a pwCF [1, 3, 4]. PEx are associated with increased morbidity, reduced quality of life, and earlier mortality for people living with chronic lung diseases, including cystic fibrosis (CF), non-CF bronchiectasis, chronic obstructive pulmonary disease (COPD), and asthma [2, 5, 6]. As PEx represent significant life events for people living with such chronic lung diseases they have been labelled as ‘lung attacks’ to call attention to their seriousness, much like a ‘heart attack’ for acute myocardial infarction [1–3]. Although the specific aetiology and underpinning mechanisms that lead to PEx are poorly understood, the most common causes of PEx in CF are by bacterial infections triggering an increased inflammatory response, leading to progressive airway damage [1, 2, 7].

While, there is no universally agreed clinical definition of PEx [1, 8], a useful simple description of PEx in CF is an acute worsening of respiratory symptoms severe enough to warrant oral or intravenous antibiotics [3]. Whereby initiation of antibiotic intervention in CF is typically based on worsening of lung function [2]; as measured by forced expiratory volume in 1 s (FEV_1_) and expressed as a normalized percent of predicted value (%FEV_1_) in a pwCF to indicate disease severity [9, 10]. Moreover, antibiotic intervention of PEx is directed against bacterial pathogens chronically infecting a pwCF [1, 2]. Prevention of PEx is one of the main goals in CF disease management [11]. Treatment of PEx typically starts when a pwCF presents as they experience increased respiratory symptoms and an acute decrease in lung function [1, 11]. However, this can lead to subsequent delays in starting antibiotic intervention and in turn could lead to more severe impact of a PEx [3, 11]. Therefore, early PEx detection and consequent timely intervention is highly desirable. Microbiological surveillance of respiratory secretions throughout the life of a pwCF is central to clinical care in CF [12, 13]. In a PEx context, culture-based surveillance is used to inform chronic infection status of canonical CF pathogens, which in turn guides choice of antimicrobial treatment [13, 14]. Plus, following PEx diagnosis, increased surveillance for the duration of treatment is recommended to further direct and assess efficacy of antibiotic therapy [12, 13]. While culture-based surveillance of targeted canonical pathogens has been useful for PEx management, it is not predictive of PEx onset and has been shown to be both limited and biased for pathogen detection in CF, e.g. [12, 15, 16].

Lower airway infections do not adhere to classical ‘one microbe, one disease’ concepts of infection pathogenesis born out of Koch’s postulates [12, 17, 18]. Instead, pulmonary infections in CF and across a range of chronic lung diseases are comprised of diverse and temporally dynamic lung infection microbiota [17–21]. Moreover, it is the case that ecological communities, whether animal, plant, or microbial, are inherently dynamic through time [22]. Wherein species constantly turnover within years, months, weeks, or even days and hours in the case of microbial species within a microbiome [22]. Yet, our understanding of human microbiota has relied heavily on spatial organization of microbial species and less on how those species are organized in time [17, 22, 23]. As such, it has been advocated that temporal dynamics set within an ecological framework need to be considered when attempting to connect change in human microbiota to change in disease status [17, 20, 24]. For example, the theory of island biogeography has proven to be a useful framework to apply to the respiratory microbiome, as the lungs can be considered as dynamic ‘island’ habitats subject to immigration and extinction of species through time [17, 25, 26]. Crucially, for understanding temporal microbiota dynamics it has temporal turnover, defined as “the number of species eliminated and replaced per unit of time”, as its central concept [17, 23].

Set within that framework and utilising species-time relationships (STRs) to measure temporal turnover, we recently determined the ecological patterns and processes underpinning temporal turnover of the chronic and intermittently infecting members of dynamic lung infection microbiota from individual children and adults with CF [17]. CF lung microbiota have high interpatient variability and are highly personalized to the individual pwCF [9], expanding upon that spatial principle we found that temporal organization, as measured with STRs, is also a highly personalized feature of the CF lung microbiome [17, 24]. We concluded that this approach incorporating a personalized patient focus has translational potential to guide and improve therapeutic targeting of lung microbiota in chronic lung diseases, including in CF.

In the current study, we applied this ecological approach to gain novel understanding of the longitudinal lung microbiota dynamics across a PEx event within individual adults with CF (awCF). Specifically, we sought to determine whether changes in bacterial species turnover within the lung microbiota of individual awCF could be indicative of PEx onset. We achieved this by retrospectively analysing longitudinal microbiota and clinical data collected from individual awCF over the course of a PEx [27]. To differentiate between disease status leading up to and after PEx in individual patients, we applied the useful ‘BETR’ disease state classifications to each individual awCF longitudinal dataset; (B) baseline, (E) exacerbation, (T) treatment, and (R) recovery [27, 28].

## Methods

### Study and patient sampling

This retrospective study utilises longitudinal data derived from a prospective study that focused on combined cohort-wide changes in lung microbiology through BETR disease periods [27]. The study was reviewed and approved by the Southampton and Southwest Hampshire Research Ethics Committee, UK (06/Q1704/24). All patients provided written informed consent. Sputum samples were collected from 12 awCF at Southampton General Hospital, Southampton, UK. Participating awCF were selected based on having experienced at least one PEx in the 12 months prior to the start of the study and were persistently sputum productive. Diagnostic culture-based microbiology indicated that all participating awCF were chronically colonised with *Pseudomonas aeruginosa* within 12 months prior to start of study, as such all participants were all receiving antibiotics with known activity against this pathogen. Patients were assessed at their usual clinic appointments by their regular clinical team and included routine and emergency visits. Spirometry was performed by their usual clinical team. The clinical characteristics of patients included in the current study are summarised in Table 1.

**Table 1:** Clinical characteristics of individual patients

			CFTR genotype^c^			Pancreatic	CF-related	PEx antibiotics^e^		PEx
Patient	Age^a^	Sex^b^	Genotype 1	Genotype 2	%FEV_1_^d^	Insufficient	diabetes	Oral	Intravenous	frequency^f^
1	30	M	F508 del	NK	57.8 ± 5.8	No	No	Ciprofloxacin		3
2	45	F	F508 del	F508 del	25.2 ± 5.6	Yes	Yes		Colistin + Tobramycin	5
3	47	M	F508 del	NK	36.5 ± 2.8	Yes	Yes			4
4	22	F	F508 del	F508 del	30.7 ± 4.9	Yes	No	Ciprofloxacin	Meropenem + Amikacin	5
5	55	M	F508 del	G58E	81.1 ± 5.0	Yes	No		Ceftazidime + Gentamicin	2
6	21	F	F508 del	F508 del	61.2 ± 6.9	Yes	No	Ciprofloxacin		3
7	40	M	F508 del	F508 del	60.8 ± 9.2	Yes	Yes			3
8	22	M	F508 del	F508 del	28.6 ± 5.6	Yes	Yes		Meropenem + Colistin	6
9	17	F	F508 del	F508 del	73.6 ± 6.5	Yes	No		Ceftazidime + Gentamicin	3
10	24	F	F508 del	G542X	64.4 ± 7.1	Yes	No	Clarithromycin		3
11	20	M	F508 del	F508 del	29.7 ± 7.8	Yes	No	Ciprofloxacin	Metronidazole	4
12	20	M	F508 del	F508 del	45.9 ± 3.3	Yes	No		Ceftazidime + Gentamicin	4

^a^Age in years

^b^Sex: *F* female and *M* male

^c^ Cystic fibrosis transmembrane conductance regulator (CFTR) genotype (NK = not known)

^d^Lung function—Mean %FEV_1_ and standard deviation of the mean (SD) over course of study

^e^Antibiotic interventions for pulmonary exacerbation (PEx) by oral or intravenous administration

^f^Number of PEx in 12 months prior to start of study

All sputum samples were stored at 4 °C immediately after spontaneous expectoration and transferred to − 80 °C storage within 12 h of production [29, 30]. Decisions to start and end treatment for PEx were directed by treating clinicians based on worsening and subsequent stabilisation or improvement of clinical symptoms, respectively. Samples and data were retrospectively partitioned into five disease periods: (B_0_) baseline pre-PEx; (E) PEx, within 30 days prior to start of antibiotic treatment; (T) period of time patients were receiving treatment for pulmonary exacerbation; (R) recovery, within 30 days post-treatment for PEx; and (B_1_) baseline post-PEx [27, 28]. During the study period 10 of the 12 awCF were treated for PEx. The two awCF (P3 and P7) that remained at baseline (B_0_) served as a useful comparison.

### Sequencing

Sputum was washed with phosphate-buffered saline to remove saliva, as previously described [31]. DNA from dead or damaged cells, as well as extracellular DNA, was excluded from analysis via cross-linking with propidium monoazide prior to DNA extraction, as previously described [32]. Subsequent DNA extraction, 16S rRNA gene targeted amplicon sequencing via barcode encoded FLX amplicon pyrosequencing, and sequence analyses within the mothur sequencing analysis platform are described in detail in Cuthbertson et al. (2016) [27]. Given the length of the ribosomal sequences analysed, species identities should be considered putative. The raw sequence data reported in this study have been deposited in the European Nucleotide Archive under accession numbers PRJEB5815 and PRJEB7346. The relevant barcode information for each sample along with anonymised clinical metadata, processed microbiota data have been deposited at figshare.com under https://figshare.com/s/f34060344dba52278861.

### Statistical analyses

All regression analyses, coefficients of determination (*R*^2^), degrees of freedom, *F*-statistics, and significance (*P*) were calculated using XLSTAT v2018.1 (Addinsoft, Paris, France). Kruskal–Wallis analyses in conjunction with the post-hoc Dunn test, were also performed in XLSTAT. Persistence-abundance relationships (PARs) were used to visualize the temporal distribution of all bacterial taxa, including chronic and intermittently colonizing taxa, within each awCF time series [17]. Using a modification of the Leeds criteria [12, 14], individual awCF were considered chronically or intermittently colonized with a given bacterial taxon if it had > 50% or ≤ 50% temporal persistence, respectively [17].

Species-time relationships (STRs) are well established ecological methods for measuring temporal turnover in communities of animals, plants, and microorganisms [17, 18]. STRs have been used to describe how the observed taxon richness of a community in a habitat of fixed size increases with the length of time over which the community is monitored [33, 34]. The STR is modelled with the power regression equation, *S* = *cT*^*w*^. Where *S* is the cumulative number of observed taxa over time *T*, *c* is the intercept, and *w* is the slope of the line or temporal scaling exponent, where increasing values of *w* can be taken as greater rates of turnover [35]. Here, STRs were used to assess species turnover within each awCF over each time series. STRs were constructed using the moving window method, as previously described [23]. Unlike other STR construction approaches, this method incorporates immigration of new species, local extinctions, and recolonizations of same taxa through time, which would be anticipated in a time series of this study’s extent [23].

To examine changes in lung function, cumulative changes in individual awCF lung function with time were calculated by taking cumulative absolute change in %FEV_1_ between consecutive timepoints across each time series. Plots of cumulative lung function change versus time were then fitted with a power regression model, Δ%FEV_1_ = *aT*^*b*^. Where Δ%FEV_1_ is cumulative change in lung function, *a* is the intercept, *T* is time in days, and *b* is the rate of change in lung function over time.

## Results

Here, we analysed respiratory samples from 12 awCF collected over the course of a PEx. The clinical characteristics of individual patients are summarised in Table 1. Two of the 12 awCF (P3 and P7) did not experience a PEx during the course of the study and hence were at baseline (B_0_) throughout. For those that did experience a PEx, the mean (± standard deviation of the mean [SD]) sample period was 315.9 ± 42.7 days (minimum = 221, maximum = 368 days). The mean (± SD) number of samples for those awCF was 22.2 ± 5.1 (minimum = 16, maximum = 35 days). For P3 and P7 the duration was 313 and 300 days, and with 9 and 6 samples collected over those periods, respectively.

### Chronic and intermittent colonization

**Fig. 1:**
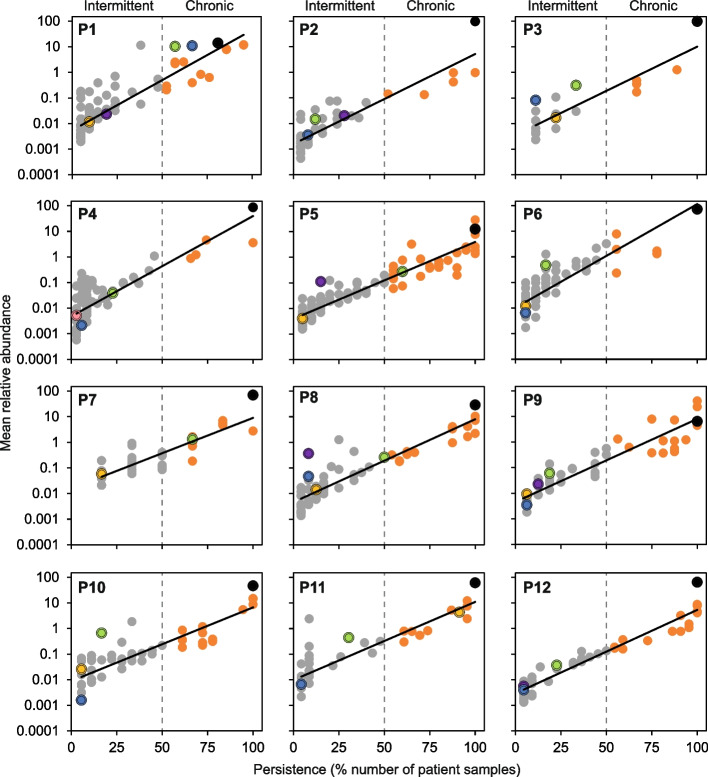
Persistence and abundance of chronic and intermittent colonizing bacterial taxa within patients. Using a modification of the Leeds criteria of chronic infection, patients were deemed to be chronically (orange circles) or intermittently (grey) colonized with a given bacterial taxon if present in > 50% or ≤ 50% samples, respectively. Canonical pathogens are marked as follows: *Pseudomonas aeruginosa*, black circle; *Staphylococcus aureus*, blue; *Stenotrophomonas maltophilia*, green; *Burkholderia cepacia* complex, pink; *Haemophilus influenzae*, purple; and *Achromobacter xylosoxidans*, yellow. Chronic or intermittent colonization status for all bacterial taxa within the microbiota of each patient is highlighted in the supplemental microbiota data (see “Data availability” section). Persistence-abundance relationship regression statistics: (P1) *R*^2^ = 0.77, *F*_1,88_ = 290.1; (P2) *R*^2^ = 0.77, *F*_1,47_ = 168.9; (P3) *R*^2^ = 0.78, *F*_1,21_ = 76.2; (P4) *R*^2^ = 0.64, *F*_1,93_ = 166.5; (P5) *R*^2^ = 0.88, *F*_1,92_ = 642.1; (P6) *R*^2^ = 0.72, *F*_1,84_ = 220.6; (P7) *R*^2^ = 0.76, *F*_1,40_ = 125.5; (P8) *R*^2^ = 0.84, *F*_1,64_ = 346.4; (P9) *R*^2^ = 0.85, *F*_1,49_ = 284.8; (P10) *R*^2^ = 0.74, *F*_1,53_ = 153.1; (P11) *R*^2^ = 0.80, *F*_1,42_ = 170.1; (P12) *R*.^2^ = 0.93, *F*_1,49_ = 675.1. All relationships were significant (*P* < 0.0001 in all instances)

To visualise the temporal distributions of every chronic and intermittently colonizing bacterial taxon within each patient, the longitudinal persistence of every bacterial taxon observed in the lung microbiota of an awCF was plotted against its mean relative abundance across the temporal samples it was detected in (Fig. 1). The resulting PARs for each patient were all positive and significant, in that chronic infecting taxa were persistent and common while the intermittent infecting taxa were infrequent and rare. The mean number (± SD) of chronic and intermittent taxa across all patients was 12.6 ± 7.0 (minimum = 5, maximum = 31) and 49.6 ± 21.4 (minimum = 18, maximum = 90), respectively (Fig. 1). All patients were chronically infected with *Pseudomonas aeruginosa*. P1 was also chronically colonized with *Staphylococcus aureus* and *Stenotrophomonas maltophilia*. P5 and P7 were also chronically colonized with the latter. While P11 was also chronically colonized with *Achromobacter xylosoxidans*. Furthermore, PARs indicated all patients were all intermittently colonized with at least one and as many as four canonical CF pathogens (Fig. 1).

### Species-time relationships and temporal turnover

**Fig. 2:**
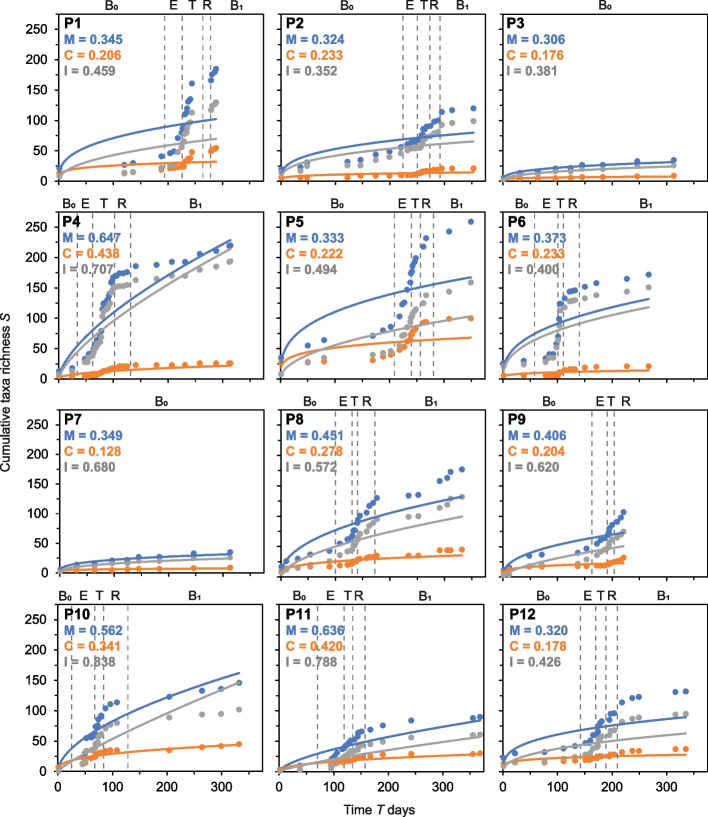
Species-time relationships within patient lung microbiota. Disease states have been superimposed for each patient: B_0_, baseline pre-exacerbation; E, exacerbation; T, treatment period; R, recovery period; and B_1_ post-exacerbation baseline. Given are species-time relationships (STRs) for microbiota (blue), and the chronically (orange) and intermittently (grey) colonising taxa in each patient (P1 to P12). Also given in each instance is the STR slope value *w* for the microbiota (M), and the chronically (C) and intermittently (I) colonising taxa. All STRs were significant (*P* < 0.05). Full STR regression statistics are reported in Table S1

Temporal turnover within the lung microbiota of each awCF was measured using the slope (*w*) values from STRs fitted to each whole-time series (Fig. 2). For each awCF, STRs for the whole microbiota as well as the chronic and intermittent colonizing taxa were plotted. The mean *w* (± SD) for the whole microbiota was 0.421 ± 0.120 (minimum = 0.306, maximum = 0.647). The mean *w* (± SD) for the chronic colonizing taxa was 0.255 ± 0.093 (minimum = 0.128, maximum = 0.438), where temporal turnover was significantly lower than observed in the whole microbiota (Kruskal–Wallis: *H* = 9.01, *P* = 0.003). Conversely, turnover in the intermittent colonizing taxa group (mean *w* = 0.560 ± 0.159 [minimum = 0.352, maximum = 0.838]) was significantly higher than in the whole microbiota (*H* = 5.88, *P* = 0.015). Turnover in the intermittent colonizing taxa was also significantly higher than in the chronic colonizing taxa group (*H* = 14.10, *P* < 0.0001). When the BETR disease state framework was superimposed to each patient time series a notable increase in cumulative taxa richness was observed in the exacerbation and treatment states for the whole microbiota and intermittent colonizing taxa, and to a lesser extent for the chronic colonizing taxa (Fig. 2). Conversely, no notable increases in taxa accumulation were observed in the awCF that did not experience a PEx during the study (P3 and P7).

### Changes in lung function with time

**Fig. 3:**
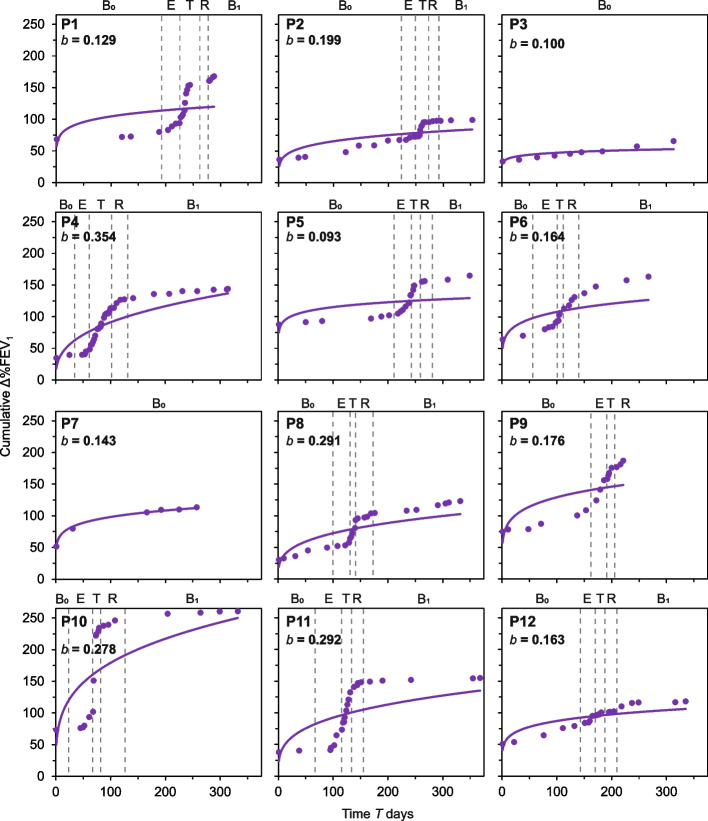
Cumulative changes in patient lung function with time. Given in each instance is the cumulative absolute change in %FEV_1_ between consecutive timepoints. Each plot has been fitted with a power regression; Δ%FEV_1_ = *aT*^b^. Where Δ%FEV_1_ is cumulative change in lung function, *a* is the intercept, *T* is time in days, and *b* = slope/rate of change in lung function over time. Disease states have been superimposed for each patient: B_0_, baseline pre-exacerbation; E, exacerbation; T, treatment period; R, recovery period; and B_1_ post-exacerbation baseline. Lung function-time relationship regression statistics: (P1) *R*^2^ = 0.25, *F*_1,19_ = 6.46, *P* = 0.02; (P2) *R*^2^ = 0.61, *F*_1,23_ = 35.60, *P* < 0.0001; (P3) *R*^2^ = 0.67, *F*_1,7_ = 14.19, *P* = 0.0007; (P4) *R*^2^ = 0.60, *F*_1,33_ = 49.22, *P* < 0.0001; (P5) *R*^2^ = 0.34, *F*_1,18_ = 9.10, *P* = 0.008; (P6) *R*^2^ = 0.51, *F*_1,16_ = 16.87, *P* = 0.001; (P7) *R*^2^ = 0.99, *F*_1,4_ = 920.53, *P* < 0.0001; (P8) *R*^2^ = 0.70, *F*_1,22_ = 50.77, *P* < 0.0001; (P9) *R*^2^ = 0.58, *F*_1,14_ = 19.25, *P* = 0.001; (P10) *R*^2^ = 0.44, *F*_1,16_ = 12.66, *P* = 0.003; (P11) *R*^2^ = 0.42, *F*_1,21_ = 15.38, *P* = 0.001; (P12) *R*.^2^ = 0.72, *F*_1,20_ = 51.99, *P* < 0.0001. All relationships were significant (*P* < 0.05 in all instances). Changes in patient lung function with time are presented in Fig. S1

Next, lung function-time relationships were plotted to investigate the cumulative absolute change in %FEV_1_ across each awCF time series (Fig. 3). Like the STRs, the cumulative absolute change in lung function (Δ%FEV_1_) over time was observed to notably increase within the exacerbation and treatment diseases states. However, notable increases in cumulative Δ%FEV_1_ were absent for the awCF that did not experience a PEx within the study (Fig. 3). Moreover, the rate of change in lung function over time (*b*) was typically higher for patients that experienced a PEx (mean *b* = 0.214 ± 0.080) when compared to those that did not (*b* for P3 and P7 was 0.100 and 0.143, respectively). Changes in %FEV_1_ through time are presented in Fig. S1.

### Relationships between changes in lung function and temporal turnover

**Fig. 4:**
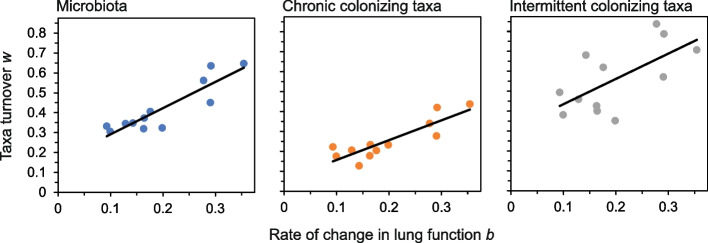
Relationship between rate of change in lung function and taxa turnover. This relationship is plotted for the whole microbiota and the chronic and intermittent colonizing taxa groups. Taxa turnover *w* is derived from species-time relationships (Fig. 2). Rate of change in lung function *b* is derived from lung function-time relationships (Fig. 3). Regression statistics: microbiota *R*^2^ = 0.80, *F*_1,10_ = 40.08, *P* < 0.0001; chronic taxa *R*^2^ = 0.76, *F*_1,10_ = 31.15, *P* < 0.0001; and Intermittent taxa *R*^2^ = 0.43, *F*_1,10_ = 7.45, *P* = 0.022. All relationships were significant at *P* < 0.05 level

When relationships between temporal turnover and lung function were explored, positive significant correlations were found between the rate of change in lung function (*b*) and STR derived turnover values (*w*) values from the whole microbiota, and the chronic and intermittent colonizing taxa groups (Fig. 4). In each instance, greater changes in lung function were accompanied with greater changes in temporal turnover (Fig. 4). Conversely, no significant relationships between mean %FEV_1_ over the course of each awCF time series and turnover (*w*) were observed (Microbiota, *F*_1,10_ = 0.99, *R*^2^ = 0.09, *P* = 0.343; Chronic, *F*_1,10_ = 0.99, *R*^2^ = 0.17, *P* = 0.188; and intermittent, *F*_1,10_ = 0.05, *R*^2^ = 0.05, *P* = 0.825).

### Rates of turnover at finer temporal scales through PEx

**Fig. 5:**
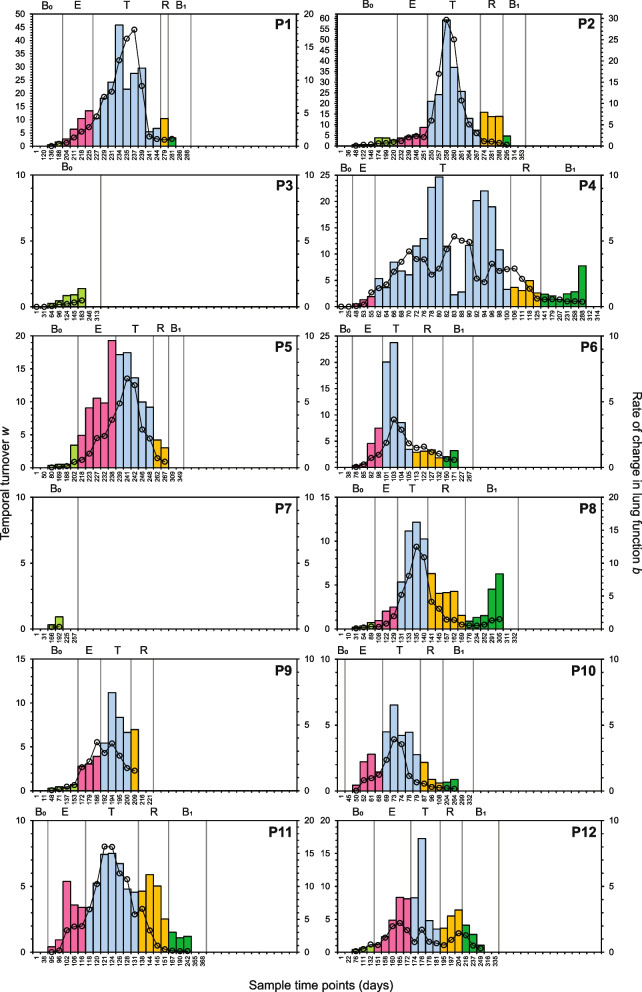
Finer scale examination of temporal turnover (*w*) and changes in rate of lung function (*b*) within individual patients. To derive finer scale measures of *w* (columns) and *b* (circles), species-time relationships and cumulative changes in lung function were respectively plotted using windows of five sample time points, shifting by one time point for each new plot along the time series for each patient (P1 to P12). Values of *w* and *b* values are plotted at the middle time point of each subsequent five time point window. Disease states have been superimposed for each patient: B_0_, baseline pre-exacerbation; E, exacerbation; T, treatment period; R, recovery period; and B_1_ post-exacerbation baseline

To further elucidate relationships between turnover, lung function, and BETR disease states through time, the temporal scale examined was changed from the entire time course to a finer scale, incorporating a moving 5 timepoint ‘quadrat’ approach. Wherein, within each awCF time series, values of *w* and *b* were calculated respectively from STRs and lung function-time relationships from the first 5 timepoints and then calculated again after moving the temporal quadrat sequentially to the second time point, then the third, and so on across the remaining time series (Fig. 5). A benefit of this more granular approach is it allowed changes across BETR disease states to be more readily determined. For brevity, we focused this analysis on turnover within the whole microbiota only. For patients who experienced a PEx, the general observed trend of microbiota turnover, albeit with substantial interpatient variation, was one of increasing values of *w* in pre-PEx baseline (B_0_, when data was available), then increasing further across exacerbation (E) and reaching a peak within treatment (T). After peaking, turnover then notably started to decrease within the treatment period, and then continued to decrease within the recovery (R), and post-PEx baseline (B_1_) disease states (Fig. 5). In some of the PEx patients (e.g. P4, P6, P8, P10, and P11) turnover was then observed to start increasing again within the B_1_ period. Within the non-PEx patients (P3 and P7) an increase in turnover was observed across time in B_0_. The rates of change in lung function (*b*) also broadly followed the observed general trend in microbiota turnover, usually with some lag and again with substantial interpatient variation (Fig. 5).

## Discussion

Acute pulmonary exacerbations (PEx) are clinically significant events that are associated with increased morbidity and earlier mortality for pwCF [8, 36]. Furthermore, PEx can account for approximately half of the loss in lung function across a pwCF’s lifetime [36]. Consequently, early detection and timely intervention is crucial [1, 2, 7]. However, PEx treatment is typically only initiated shortly after a pwCF presents with worsening respiratory symptoms [9, 17]. Hence, there is a pressing need to identify predictive biomarkers of PEx onset [2, 8, 36]. Here, we investigated temporal turnover to connect longitudinal changes within the lung microbiota of individual awCF to changes in disease states across a PEx event. Despite the highly personalized nature of CF lung microbiota, we found proof of concept that changes in temporal turnover could be indicative of the onset of PEx.

In agreement with our previous work, that first devised a modification of the Leeds criteria to define chronic infection status for all members of the microbiota within individual pwCF, we found that individual patients can be chronically colonized with not just a single pathogen but with multiple bacterial species, including multiple canonical CF pathogens (Fig. 1) [17]. In contrast, culture-based microbiological data for the study patients indicated that each patient was chronically colonized only with *Pseudomonas aeruginosa*. Culture offers a limited surveillance assessment that is constrained to detecting presence or absence of a handful of targeted bacteria within a respiratory sample [12]. Thus, not only does it provide a blinkered view of the microbial species present, but it is also now recognized that culture can significantly underestimate presence of readily cultivable pathogens, including *P. aeruginosa* and *S. aureus* [12, 17]. Combined this has implications for choosing optimal antimicrobial interventions in general as well as to combat PEx [17, 30]. This adds further weight to recommending use of molecular approaches for microbiological surveillance in CF that define all microbial species present within an individual’s lung infection microbiota through time [12, 17].

For temporal turnover, high interpatient variability was evident in both the STR *w*-values and within the shapes of individual STRs for the whole microbiota and the chronic and intermittent colonizing groups (Fig. 2). This indicated that turnover and hence temporal organization are a personalized feature of the lung microbiota in CF, adding further support to the premise that CF lung microbiota through time and space are highly personalized to the individual pwCF [17, 19, 20, 37]. An expectation of STRs where no system perturbation occurs would be relatively low *w*-values and a steady cumulation of species richness through time [38]. This was observed for the STRs for the awCF that did not experience a PEx (Fig. 2). Conversely, those that did experience a PEx all demonstrated typically higher *w*-values in comparison along with notable increases in species richness cumulation through both the exacerbation and treatment periods (Fig. 2). Interestingly, the lung function-time relationships also exhibited similar patterns of increases in cumulative Δ%FEV_1_ and hence that was also highly personalized to the individual pwCF (Fig. 3). Moreover, significant positive relationships were observed between values for rates of change in lung function (*b*) and turnover (*w*) within the microbiota, and chronic and intermittent colonizing taxa. This revealed that greater turnover was associated with greater change in lung function with time (Fig. 4). An explanation for the personalized nature of these observations could stem from that CF PEx events are likely not all the same between and even within pwCF [39]. Wherein, PEx are now thought to be caused by intricate and still not fully understood interactions between the patient and bacteria [1], fungi [40], respiratory viruses [41], and air pollution [42] either singularly or in combination, further compounding the heterogenous nature of PEx.

When microbiota turnover was examined at a finer scale across the time series for each awCF, values of *w* could clearly be observed to increase in the exacerbation period and often from within the pre-PEx baseline period and peaking within the treatment period (Fig. 5). This demonstrated that increases in turnover were not solely the result of the start of treatment and subsequent PEx antibiotic administration causing perturbations to the lung microbiome. Consequently, we posit that the increase in turnover ahead of treatment could be indicative of the onset of a PEx. Meta-analyses of STRs across different lengths of time and covering communities of animals, plants, and microbes across a wide variety of habitats and ecosystems have found STR derived rates of turnover (*w*) to have a remarkable degree of regularity that typically do not exceed more than 0.5 [43, 44]. Here, we found *w*-values soon increased beyond that, with a mean peak *w* (± SD) across PEx patients of 22.74 ± 16.28 (minimum *w* = 6.53, maximum *w* = 59.21) (Fig. 5). As such, it was possible to use the departure from normal ranges of *w* up to the initiation of treatment to approximate onset of PEx in days across the awCF in the study. From *w* exceeding the typical maximum of *w* = 0.5 to start of treatment initiation the mean (± SD) was 35.8 ± 20.1 days across PEx patients. When we take a more parsimonious approach and conservatively increase the threshold to *w* > 1.0, to account for any negligible departure from the expected norm, the onset of exacerbation to start of treatment becomes 21.2 ± 8.9 days. Additionally, retrospective checking of clinical records, revealed for the PEx patients which had observed increases in turnover within the post-PEx baseline period (e.g. P4, P6, P8, P10, and P11) and the two non-PEx patients who both experienced increases in turnover across baseline (B_0_), all went onto experience a PEx and intervention for PEx within weeks after the end of the study sampling. Regardless of which cutoff of turnover (*w* > 0.5 or *w* > 1.0) is used to indicate the onset of PEx, it is fair to state that both provide a considerable lead in before the start of antibiotic treatment. Therefore, our findings offer an exciting proof of concept which uniquely establishes that changes in microbiota turnover could potentially be used to indicate onset of an approaching PEx. Combined with incorporation of molecular approaches that define all microbial species present into microbiological surveillance of respiratory secretions throughout the life of a pwCF, this could enable an opportunity for pre-emptive intervention, which in turn could reduce the impacts of a PEx both in the short- and cumulatively in the long-term that result from delayed antibiotic interventions.

There are potential limitations to this study that deserve consideration. While our work offers an exciting prospect to identify onset of PEx, to further advance this work future clinical studies will need to have larger patient numbers representative of the wider CF population, including adults and children with CF. It is the case that more granular and longer longitudinal microbiota studies in CF are accompanied by an inevitable reduction in the amount of participating pwCF, as was the case here [17, 19, 20, 37]. While these ecology-based studies are providing previously unrealised and hard-won insight into the temporal dynamics of lung infection microbiota, planning of future clinical studies will need to carefully consider how to maximise patient participation and retention. Samples underpinning the data analysed in this retrospective study were collected before widespread availability of effective CF transmembrane conductance regulator (CFTR) modulators (e.g. elexacaftor/tezacaftor/ivacaftor [ETI]). How the temporal dynamics of lung infection microbiota will be affected within people on CFTR modulators is not well understood [17, 37]. Moreover, although ETI therapy has been shown to reduce the frequency of PEx, they still occur and are clinically impactful [45, 46]. Further, due to significant improvements in lung function experienced by pwCF on ETI therapy [46], alternatives to %FEV_1_ as a clinical indicator of health in CF are required. Additionally, in countries such as the UK and USA, approximately 10% of the CF population either do not have eligible CFTR mutations or do not tolerate CFTR modulator therapy [47]. Likewise, there are many countries where there is limited or no access to CFTR modulator therapies [47]. Therefore, for those pwCF who are ineligible, intolerant, or lack access to modulators our findings remain directly relevant.

## Conclusions

Setting microbiome research within a theoretical ecology framework is a parsimonious and pragmatic solution to understanding and predicting the complex ecology of respiratory microbiota related to clinical outcomes [25, 48]. The application of ecological theory to microbiome research is currently very limited [17, 18]. However, the power of these approaches are indeed becoming increasingly apparent in respiratory microbiome research, from using ecological modelling to define distinct PEx microbiota types and predicting treatment outcomes for PEx [20], to here where we illustrate that changes in temporal turnover are indicative of PEx onset in considerable advance of when antimicrobial treatment would normally be initiated. These theoretical based advances are all underpinned by considering temporal microbiome dynamics to connect changes in human microbiota to change in health state [17, 20, 37]. This goes beyond what traditional ‘one microbe, one disease’ approaches alone are capable of when combatting polymicrobial infections in chronic lung diseases [17]. Application of our work offers novel translational potential to enable early detection of PEx in CF and hence could allow consequent timely intervention and improve PEx management and even prevention. Moving on from basic science research, future clinical studies with greater patient numbers will be needed to realise this exciting potential. There is also real possibility to apply the temporal framework used here to PEx events across a range of chronic airway diseases.

## Supplementary Information

Additional file 1: Supplementary Table S1. Species-time relationship (STR) regression statistics. Given are slope *w* and intercept *c* from the STR power function. Also given in each instance are degrees of freedom (df), *F*-statistic, coefficient of determination (*R*^2^), and significance (*P*).

Additional file 2: Supplementary Figure S1. Changes in patient lung function with time. Given in each instance is the %FEV_1_ for consecutive timepoints. Disease states have been superimposed for each patient: B_0_, baseline pre-exacerbation; E, exacerbation; T, treatment period; R, recovery period; and B_1_ post-exacerbation baseline.

## Data Availability

The raw sequence data reported in this study have been deposited in the European Nucleotide Archive under accession numbers PRJEB581 and PRJEB7346. Anonymised clinical metadata, processed microbiota data have been deposited at figshare.com under https://figshare.com/s/f34060344dba52278861.

## References

[1] Bhatt JM. Treatment of pulmonary exacerbations in cystic fibrosis. Eur Respir Rev. 2013;22:205. 10.1183/09059180.00006512. 23997047 10.1183/09059180.00006512 PMC9487366

[2] Stanford GE, Dave K, Simmonds NJ. Pulmonary exacerbations in adults with cystic fibrosis: a grown-up issue in a changing cystic fibrosis landscape. Chest. 2021;159:93-102. 10.1016/j.chest.2020.09.084. 32966813 10.1016/j.chest.2020.09.084 PMC7502225

[3] de Boer K, Vandemheen KL, Tullis E, Doucette S, Fergusson D, Freitag A, et al. Exacerbation frequency and clinical outcomes in adult patients with cystic fibrosis. Thorax. 2011;66:680-5. 10.1136/thx.2011.161117. 21680566 10.1136/thx.2011.161117

[4] FitzGerald JM. Targeting lung attacks. Thorax. 2011;66:365-6. 10.1136/thx.2010.156760. 21398372 10.1136/thx.2010.156760

[5] Castillo JR, Peters SP, Busse WW. Asthma exacerbations: pathogenesis, prevention, and treatment. J Allergy Clin Immunol Pract. 2017;5:918-27. 10.1016/j.jaip.2017.05.001. 28689842 10.1016/j.jaip.2017.05.001 PMC5950727

[6] Viniol C, Vogelmeier CF. Exacerbations of COPD. Eur Respir Rev. 2018;27:170103. 10.1183/16000617.0103-2017. 29540496 10.1183/16000617.0103-2017 PMC9488662

[7] Goss CH. Acute pulmonary exacerbation in cystic fibrosis. Semin Respir Crit Care Med. 2019;40:792–803. 10.1055/s-0039-1697975. 31659730 10.1055/s-0039-1697975 PMC7528649

[8] Almulhem M, Ward C, Haq I, Gray RD, Brodlie M. Definitions of pulmonary exacerbation in people with cystic fibrosis: a scoping review. BMJ Open Respir Res. 2024;11:e002456. 10.1136/bmjresp-2024-002456. 39147400 10.1136/bmjresp-2024-002456 PMC11331921

[9] Cuthbertson L, Walker AW, Oliver AE, Rogers GB, Rivett DW, Hampton TH, et al. Lung function and microbiota diversity in cystic fibrosis. Microbiome. 2020;8:45. 10.1186/s40168-020-00810-3. 32238195 10.1186/s40168-020-00810-3 PMC7114784

[10] Quanjer PH, Stanojevic S, Cole TJ, Baur X, Hall GL, Culver BH, et al. Multi-ethnic reference values for spirometry for the 3–95-yr age range: the global lung function 2012 equations. Eur Respir J. 2012;40:1324. 10.1183/09031936.00080312. 22743675 10.1183/09031936.00080312 PMC3786581

[11] van Horck M, Winkens B, Wesseling G, van Vliet D, van de Kant K, Vaassen S, et al. Early detection of pulmonary exacerbations in children with Cystic Fibrosis by electronic home monitoring of symptoms and lung function. Sci Rep. 2017;7:12350. 10.1038/s41598-017-10945-3. 28955051 10.1038/s41598-017-10945-3 PMC5617859

[12] Gavillet H, Hatfield L, Rivett D, Jones A, Maitra A, Horsley A, et al. Bacterial culture underestimates lung pathogen detection and infection status in cystic fibrosis. Microbiol Spectr. 2022;10:e00419-22. 10.1128/spectrum.00419-22. 35972283 10.1128/spectrum.00419-22 PMC9602735

[13] Smyth AR, Bell SC, Bojcin S, Bryon M, Duff A, Flume P, et al. European Cystic Fibrosis Society standards of care: best practice guidelines. J Cyst Fibros. 2014;13:S23-S42. 10.1016/j.jcf.2014.03.010. 24856775 10.1016/j.jcf.2014.03.010

[14] Lee TWR, Brownlee KG, Conway SP, Denton M, Littlewood JM. Evaluation of a new definition for chronic Pseudomonas aeruginosa infection in cystic fibrosis patients. J Cyst Fibros. 2003;2:29-34. 10.1016/S1569-1993(02)00141-8. 15463843 10.1016/S1569-1993(02)00141-8

[15] Hughes DA, Rosenthal M, Cuthbertson L, Ramadan N, Felton I, Simmonds NJ, et al. An invisible threat? Aspergillus positive cultures and co-infecting bacteria in airway samples. J Cyst Fibros. 2022. 10.1016/j.jcf.2022.07.009. 35871975 10.1016/j.jcf.2022.07.009

[16] Zirbes CF, Pitcher NJ, Davis JC, Bartels AR, Krogh JD, Teresi M, et al. Staphylococcus aureus detection from CF respiratory samples is improved using alternative media. J Cyst Fibros. 2022;21:888-9. 10.1016/j.jcf.2022.04.017. 35491319 10.1016/j.jcf.2022.04.017 PMC10152491

[17] Gavillet H, Hatfield L, Jones A, Maitra A, Horsley A, Rivett D, et al. Ecological patterns and processes of temporal turnover within lung infection microbiota. Microbiome. 2024;12:63. 10.1186/s40168-024-01780-6. 38523273 10.1186/s40168-024-01780-6 PMC10962200

[18] Rivett DW, Hatfield LR, Gavillet H, Hardman M, van der Gast C. Bacterial interactions underpin worsening lung function in cystic fibrosis-associated infections. mBio. 2024;0:e01456-24. 10.1128/mbio.01456-24. 10.1128/mbio.01456-24 PMC11708055 39576107

[19] Raghuvanshi R, Vasco K, Vázquez-Baeza Y, Jiang L, Morton James T, Li D, et al. High-resolution longitudinal dynamics of the cystic fibrosis sputum microbiome and metabolome through antibiotic therapy. mSystems. 2020;5:e00292-20. 10.1128/mSystems.00292-20. 10.1128/mSystems.00292-20 PMC7311317 32576651

[20] Widder S, Carmody LA, Opron K, Kalikin LM, Caverly LJ, LiPuma JJ. Microbial community organization designates distinct pulmonary exacerbation types and predicts treatment outcome in cystic fibrosis. Nat Commun. 2024;15:4889. 10.1038/s41467-024-49150-y. 38849369 10.1038/s41467-024-49150-y PMC11161516

[21] Hampton TH, Thomas D, van der Gast C, O’Toole GA, Stanton BA. Mild cystic fibrosis lung disease is associated with bacterial community stability. Microbiol Spectr. 2021;9:10.1128/spectrum.00029-21. 10.1128/spectrum.00029-21. 10.1128/spectrum.00029-21 PMC8549546 34232099

[22] Yin H, Rudolf VHW. Time is of the essence: a general framework for uncovering temporal structures of communities. Ecol Lett. 2024;27:e14481. 10.1111/ele.14481. 39022847 10.1111/ele.14481

[23] Rivett DW, Mombrikotb SB, Gweon HS, Bell T, van der Gast C. Bacterial communities in larger islands have reduced temporal turnover. ISME J. 2021;15:2947-55. 10.1038/s41396-021-00976-0. 33941889 10.1038/s41396-021-00976-0 PMC8443627

[24] Flores GE, Caporaso JG, Henley JB, Rideout JR, Domogala D, Chase J, et al. Temporal variability is a personalized feature of the human microbiome. Genome Biol. 2014;15:531. 10.1186/s13059-014-0531-y. 25517225 10.1186/s13059-014-0531-y PMC4252997

[25] Dickson RP, Erb-Downward JR, Huffnagle GB. Towards an ecology of the lung: new conceptual models of pulmonary microbiology and pneumonia pathogenesis. Lancet Respir Med. 2014;2:238-46. 10.1016/S2213-2600(14)70028-1. 24621685 10.1016/S2213-2600(14)70028-1 PMC4004084

[26] Whiteson KL, Bailey B, Bergkessel M, Conrad D, Delhaes L, Felts B, et al. The upper respiratory tract as a microbial source for pulmonary infections in cystic fibrosis. parallels from island biogeography. Am J Respir Crit Care Med. 2014;189:1309-15. 10.1164/rccm.201312-2129PP. 10.1164/rccm.201312-2129PP PMC4098084 24702670

[27] Cuthbertson L, Rogers GB, Walker AW, Oliver A, Green LE, Daniels TWV, et al. Respiratory microbiota resistance and resilience to pulmonary exacerbation and subsequent antimicrobial intervention. ISME J. 2016;10:1081-91. 10.1038/ismej.2015.198. 26555248 10.1038/ismej.2015.198 PMC4820042

[28] Zhao J, Schloss PD, Kalikin LM, Carmody LA, Foster BK, Petrosino JF, et al. Decade-long bacterial community dynamics in cystic fibrosis airways. Proc Natl Acad Sci USA. 2012;109:5809-14. 10.1073/pnas.1120577109. 22451929 10.1073/pnas.1120577109 PMC3326496

[29] Cuthbertson L, Rogers GB, Walker AW, Oliver A, Hoffman LR, Carroll MP, et al. Implications of multiple freeze-thawing on respiratory samples for culture-independent analyses. J Cyst Fibrosis. 2015;14:464-7. 10.1016/j.jcf.2014.10.004. 10.1016/j.jcf.2014.10.004 PMC4793934 25459563

[30] Cuthbertson L, Rogers Geraint B, Walker Alan W, Oliver A, Hafiz T, Hoffman Lucas R, et al. Time between collection and storage significantly influences bacterial sequence composition in sputum samples from cystic fibrosis respiratory infections. J Clin Microbiol. 2020;52:3011-6. 10.1128/jcm.00764-14. 10.1128/JCM.00764-14 PMC4136140 24920767

[31] Rogers GB, Carroll MP, Serisier DJ, Hockey PM, Jones G, Kehagia V, et al. Use of 16S rRNA gene profiling by terminal restriction fragment length polymorphism analysis to compare bacterial communities in sputum and mouthwash samples from patients with cystic fibrosis. J Clin Microbiol. 2006;44:2601-4. 10.1128/jcm.02282-05. 16825392 10.1128/JCM.02282-05 PMC1489498

[32] Rogers GB, Cuthbertson L, Hoffman LR, Wing PAC, Pope C, Hooftman DAP, et al. Reducing bias in bacterial community analysis of lower respiratory infections. ISME J. 2013;7:697-706. 10.1038/ismej.2012.145. 23190732 10.1038/ismej.2012.145 PMC3603400

[33] Oliver AE, Newbold LK, Whiteley AS, van der Gast CJ. Marine bacterial communities are resistant to elevated carbon dioxide levels. Environ Microbiol Rep. 2014;6:574-82. 10.1111/1758-2229.12159. 25756110 10.1111/1758-2229.12159

[34] van der Gast CJ, Ager D, Lilley AK. Temporal scaling of bacterial taxa is influenced by both stochastic and deterministic ecological factors. Environ Microbiol. 2008;10:1411-8. 10.1111/j.1462-2920.2007.01550.x. 18205822 10.1111/j.1462-2920.2007.01550.x

[35] Rogers GB, Skelton S, Serisier DJ, van der Gast CJ, Bruce KD. Determining cystic fibrosis-affected lung microbiology: comparison of spontaneous and serially induced sputum samples by use of terminal restriction fragment length polymorphism profiling. J Clin Microbiol. 2010;48:78-86. 10.1128/jcm.01324-09. 19906901 10.1128/JCM.01324-09 PMC2812251

[36] Singh A, He M, Chen V, Hollander Z, Tebbutt SJ, Ng RT, et al. Blood biomarkers to predict short-term pulmonary exacerbation risk in children and adolescents with CF: a pilot study. J Cyst Fibros. 2020;19:49-51. 10.1016/j.jcf.2019.05.020. 31176669 10.1016/j.jcf.2019.05.020

[37] Martin C, Guzior DV, Gonzalez CT, Okros M, Mielke J, Padillo L, et al. Longitudinal microbial and molecular dynamics in the cystic fibrosis lung after Elexacaftor–Tezacaftor–Ivacaftor therapy. Respir Res. 2023;24:317. 10.1186/s12931-023-02630-z. 38104128 10.1186/s12931-023-02630-z PMC10725582

[38] Ager D, Evans S, Li H, Lilley AK, van der Gast CJ. Anthropogenic disturbance affects the structure of bacterial communities. Environ Microbiol. 2010;12:670-8. 10.1111/j.1462-2920.2009.02107.x. 20002134 10.1111/j.1462-2920.2009.02107.x

[39] Carter SC, Franciosi AN, O’Shea KM, O’Carroll OM, Sharma A, Bell A, et al. Acute pulmonary exacerbation phenotypes in patients with cystic fibrosis. Ann Am Thorac Soc. 2022;19:1818-26. 10.1513/AnnalsATS.202111-1266OC. 35713619 10.1513/AnnalsATS.202111-1266OC PMC9667812

[40] Amin R, Dupuis A, Aaron SD, Ratjen F. The effect of chronic infection with Aspergillus fumigatus on lung function and hospitalization in patients with cystic fibrosis. Chest. 2010;137:171-6. 10.1378/chest.09-1103. 19567494 10.1378/chest.09-1103

[41] Thornton CS, Caverly LJ, Kalikin LM, Carmody LA, McClellan S, LeBar W, et al. Prevalence and clinical impact of respiratory viral infections from the STOP2 study of cystic fibrosis pulmonary exacerbations. Ann Am Thorac Soc. 2024;21:595-603. 10.1513/AnnalsATS.202306-576OC. 37963297 10.1513/AnnalsATS.202306-576OC PMC10995546

[42] Goeminne PC, Kiciński M, Vermeulen F, Fierens F, De Boeck K, Nemery B, et al. Impact of air pollution on cystic fibrosis pulmonary exacerbations: a case-crossover analysis. Chest. 2013;143:946-54. 10.1378/chest.12-1005. 23081770 10.1378/chest.12-1005

[43] Shade A, Gregory Caporaso J, Handelsman J, Knight R, Fierer N. A meta-analysis of changes in bacterial and archaeal communities with time. ISME J. 2013;7:1493-506. 10.1038/ismej.2013.54. 23575374 10.1038/ismej.2013.54 PMC3721121

[44] White EP, Adler PB, Lauenroth WK, Gill RA, Greenberg D, Kaufman DM, et al. A comparison of the species-time relationship across ecosystems and taxonomic groups. Oikos. 2006;112:185-95.

[45] Cogen JD, Quon BS. Update on the diagnosis and management of cystic fibrosis pulmonary exacerbations. J Cyst Fibros. 2024;23:603-11. 10.1016/j.jcf.2024.04.004. 38677887 10.1016/j.jcf.2024.04.004

[46] Sutharsan S, Dillenhoefer S, Welsner M, Stehling F, Brinkmann F, Burkhart M, et al. Impact of elexacaftor/tezacaftor/ivacaftor on lung function, nutritional status, pulmonary exacerbation frequency and sweat chloride in people with cystic fibrosis: real-world evidence from the German CF Registry. Lancet Reg Health Eur. 2023;32. 10.1016/j.lanepe.2023.100690. 10.1016/j.lanepe.2023.100690 PMC10405057 37554663

[47] Kramer-Golinkoff E, Camacho A, Kramer L, Taylor-Cousar JL. A survey: Understanding the health and perspectives of people with CF not benefiting from CFTR modulators. Pediatr Pulmonol. 2022;57:1253-61. 10.1002/ppul.25859. 35170259 10.1002/ppul.25859 PMC9314897

[48] Kramer-Golinkoff E, Camacho A, Kramer L, Taylor-Cousar JL. A survey: Understanding the health and perspectives of people with CF not benefiting from CFTR modulators. Pediatr Pulmonol. 2022;57:1253-61. 10.1038/nrmicro1643. 35170259 10.1002/ppul.25859 PMC9314897

