# Supplementary material for: Species turnover within cystic fibrosis lung microbiota is indicative of acute pulmonary exacerbation onset
Source: Microbiome. 2025 Jun 7;13:140. doi: 10.1186/s40168-025-02143-5 (PMC12144788; doi:10.1186/s40168-025-02143-5)
Supplement: Supplementary file 2 — Additional file 1: Supplementary Table S1. Species-time relationship (STR) regression statistics. Given are slope w and intercept c from the STR power function. Also given in each instance are degrees of freedom (df), F-statistic, coefficient of determination (R2), and significance (P). [file 40168_2025_2143_MOESM1_ESM.docx]

**Table S1** Species-time relationship (STR) regression statistics. Given are slope *w* and intercept *c* from the STR power function. Also given in each instance are degrees of freedom (df), *F*-statistic, coefficient of determination (*R*^2^), and significance (*P*)

| Patient |  | *w* | *c* | df | *F* | *R*^2^ | *P* |
| --- | --- | --- | --- | --- | --- | --- | --- |
| P1 | Microbiota | 0.345 | 14.47 | 1,19 | 11.65 | 0.38 | 0.003 |
|  | Chronic taxa | 0.206 | 5.17 | 1,19 | 7.99 | 0.30 | 0.011 |
|  | Intermittent taxa | 0.459 | 9.99 | 1,19 | 14.73 | 0.44 | 0.001 |
| P2 | Microbiota | 0.324 | 11.94 | 1,23 | 35.06 | 0.61 | <0.0001 |
|  | Chronic taxa | 0.233 | 3.70 | 1,23 | 18.25 | 0.48 | <0.0001 |
|  | Intermittent taxa | 0.352 | 8.31 | 1,23 | 40.27 | 0.64 | <0.0001 |
| P3 | Microbiota | 0.306 | 5.43 | 1,7 | 164.27 | 0.95 | <0.0001 |
|  | Chronic taxa | 0.176 | 2.76 | 1,7 | 45.28 | 0.74 | <0.0001 |
|  | Intermittent taxa | 0.381 | 2.74 | 1,7 | 272.49 | 0.97 | <0.0001 |
| P4 | Microbiota | 0.647 | 5.55 | 1,33 | 73.61 | 0.75 | <0.0001 |
|  | Chronic taxa | 0.438 | 1.77 | 1,33 | 35.17 | 0.73 | <0.0001 |
|  | Intermittent taxa | 0.707 | 3.67 | 1,33 | 87.21 | 0.77 | <0.0001 |
| P5 | Microbiota | 0.333 | 23.89 | 1,18 | 23.82 | 0.57 | <0.0001 |
|  | Chronic taxa | 0.222 | 18.49 | 1,18 | 11.57 | 0.65 | <0.0001 |
|  | Intermittent taxa | 0.494 | 5.72 | 1,18 | 55.39 | 0.43 | <0.0001 |
| P6 | Microbiota | 0.373 | 16.37 | 1,16 | 13.05 | 0.58 | 0.002 |
|  | Chronic taxa | 0.233 | 3.79 | 1,16 | 6.48 | 0.43 | 0.022 |
|  | Intermittent taxa | 0.400 | 12.62 | 1,16 | 14.11 | 0.59 | 0.002 |
| P7 | Microbiota | 0.348 | 0.64 | 1,4 | 16.25 | 0.94 | 0.016 |
|  | Chronic taxa | 0.128 | 7.26 | 1,4 | 13.61 | 0.77 | 0.021 |
|  | Intermittent taxa | 0.681 | 6.06 | 1,4 | 24.09 | 0.86 | 0.008 |
| P8 | Microbiota | 0.451 | 9.66 | 1,22 | 84.91 | 0.79 | <0.0001 |
|  | Chronic taxa | 0.278 | 7.11 | 1,22 | 63.94 | 0.74 | <0.0001 |
|  | Intermittent taxa | 0.572 | 3.59 | 1,22 | 108.35 | 0.83 | <0.0001 |
| P9 | Microbiota | 0.406 | 8.15 | 1,14 | 80.89 | 0.85 | <0.0001 |
|  | Chronic taxa | 0.204 | 7.55 | 1,14 | 60.80 | 0.81 | <0.0001 |
|  | Intermittent taxa | 0.620 | 1.79 | 1,14 | 99.95 | 0.88 | <0.0001 |
| P10 | Microbiota | 0.562 | 6.22 | 1,16 | 89.58 | 0.85 | <0.0001 |
|  | Chronic taxa | 0.341 | 6.10 | 1,16 | 63.37 | 0.80 | <0.0001 |
|  | Intermittent taxa | 0.838 | 1.13 | 1,16 | 140.88 | 0.90 | <0.0001 |
| P11 | Microbiota | 0.636 | 1.96 | 1,21 | 90.53 | 0.83 | <0.0001 |
|  | Chronic taxa | 0.420 | 2.37 | 1,21 | 129.16 | 0.83 | <0.0001 |
|  | Intermittent taxa | 0.788 | 5.45 | 1,21 | 65.36 | 0.81 | <0.0001 |
| P12 | Microbiota | 0.320 | 13.96 | 1,20 | 25.87 | 0.59 | <0.0001 |
|  | Chronic taxa | 0.178 | 9.80 | 1,20 | 21.14 | 0.53 | <0.0001 |
|  | Intermittent taxa | 0.426 | 5.26 | 1,20 | 32.39 | 0.64 | <0.0001 |
