# Supplementary material for: Species turnover within cystic fibrosis lung microbiota is indicative of acute pulmonary exacerbation onset
Source: Microbiome. 2025 Jun 7;13:140. doi: 10.1186/s40168-025-02143-5 (PMC12144788; doi:10.1186/s40168-025-02143-5)
Supplement: Supplementary file 3 — Additional file 2: Supplementary Figure S1. Changes in patient lung function with time. Given in each instance is the %FEV1 for consecutive timepoints. Disease states have been superimposed for each patient: B0, baseline pre-exacerbation; E, exacerbation; T, treatment period; R, recovery period; and B1 post-exacerbation baseline. [file 40168_2025_2143_MOESM2_ESM.docx]

**
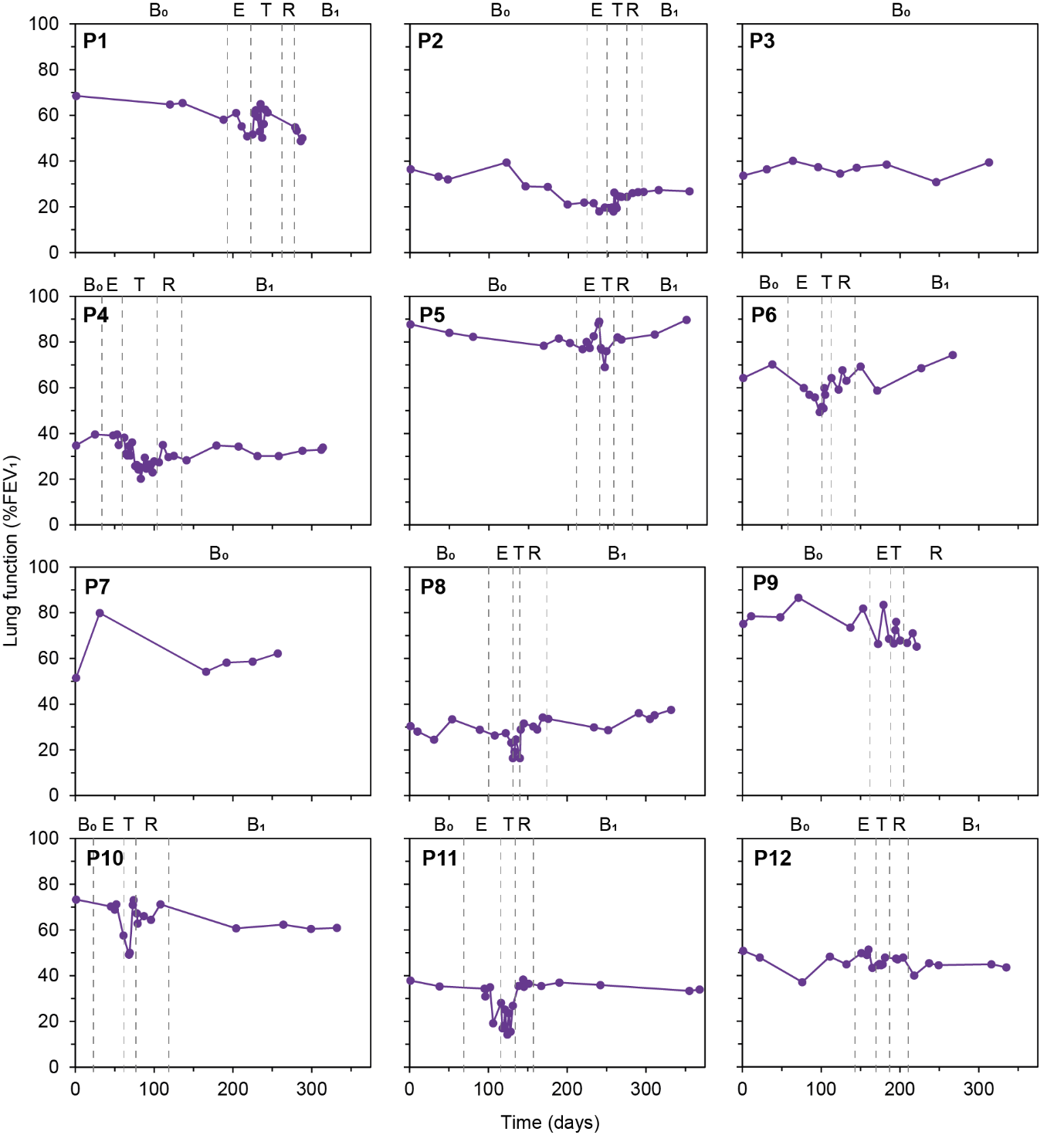
**

**Figure S1** **Changes in patient lung function with time.** Given in each instance is the %FEV_1_ for consecutive timepoints. Disease states have been superimposed for each patient: B_0_, baseline pre-exacerbation; E, exacerbation; T, treatment period; R, recovery period; and B_1_ post-exacerbation baseline.
